# Long-term effects on the quality of life following cochlear implant treatment in older patients

**DOI:** 10.1007/s00405-022-07354-2

**Published:** 2022-04-02

**Authors:** Christian Issing, Svea Holtz, Andreas G. Loth, Uwe Baumann, Johannes Pantel, Timo Stöver

**Affiliations:** 1 Department of Oto-Rhino-Laryngology, University Hospital Frankfurt, Goethe University Frankfurt/Main, Frankfurt am Main, Germany; 2 Section of Geriatric Medicine with focus on Psychogeriatrics and Clinical Gerontology, Institute of General Practice, Goethe University Frankfurt/Main, Frankfurt am Main, Germany

**Keywords:** Cochlear implant, Quality of life, Elderly, Older patients, Long-term results

## Abstract

**Purpose:**

Even in older patients, hearing rehabilitation with a cochlear implant has become an established method for deafened or severely hearing-impaired patients. In addition to the hearing improvement, numerous other effects of CI treatment can be observed in clinical routine. In the literature, there is multiple evidence for a rapid and significant improvement in quality of life with CI treatment. The aim of this study was to evaluate the long-term effects of hearing rehabilitation using CI on the quality of life in older patients (≥ 65 years).

**Methods:**

This prospective cross-sectional study examined 84 patients between the age of 65 and 101 years who received unilateral CI treatment for the first time between one and 10 years ago. The World Health Organization Quality-of-Life Scale-Old (WHOQL-OLD) was used to determine the quality of life. The study cohort was divided into three groups to compare the quality of life over time: group I (1–3 years after CI treatment), group II (4–6 years after CI treatment), and group III (7–10 years after CI treatment). In addition, the data from this study were compared with the results of our previous study (Issing et al. 2020) in which we focused on the first 6 months after CI treatment.

**Results:**

In all three groups, there was a significant improvement in monosyllabic discrimination within 1 year after CI fitting (*p* > 0.001). No significant differences were found between the three groups. There were no significant differences between the three groups in the WHOQOL-OLD total score (*p* = 0.487) or any of the other six facets. Moreover, no significant differences were found compared to the study group of our previous study 6 months after CI treatment.

**Conclusion:**

This study demonstrates the long-term stability of the improved quality of life following unilateral CI treatment in patients aged 65 years or older.

## Introduction

Due to the demographic development in the western industrialized nations, the proportion of elderly patients is continuously rising. In the elderly population, severe hearing loss or deafness is one of the most common chronic conditions [1–4]. Consequently, the proportion of cochlear implant candidates over 65 years represents a considerable percentage of the patients treated in cochlear implant (CI) centers.

For more than three decades, CI have been used successfully for functional hearing rehabilitation of severely hearing-impaired and deaf patients [5–7]. Previous studies have confirmed a significant gain in speech understanding also in the elderly [5, 7–11]. Consequently, CI treatment is performed in patients in many countries without an age limitation.

The success of hearing rehabilitation is measured primarily by audiological criteria—especially with speech understanding. In recent years, clinical research has increasingly focused on the effects of CI treatment in elderly patients beyond the improvement of speech understanding, as several studies have demonstrated a positive effect of hearing rehabilitation on the cognitive functions of elderly patients [12–16].

For the patient, the most important measure besides the success of hearing rehabilitation is the improvement in quality of life. Hearing deprivation often leads to social isolation and, as a result, to a massive reduction in the quality of life of elderly patients [17–19].

In the literature, there is several evidence for a timely and substantial improvement in quality of life with CI treatment in elderly patients [4, 18, 20–23]. Previous studies have focused on suitable measurement instruments for evaluating the quality of life in elderly patients, the speed of effects, and possible correlations. The focus of our previous study was primarily on the short- and medium-term development of quality of life after CI treatment. Therefore, this prospective study aimed to evaluate the long-term effects on the quality of life after hearing rehabilitation with CI in elderly patients.

## Patients and methods

### Study design

This prospective cross-sectional study was conducted at the Department of Oto-Rhino-Laryngology, Medical University Frankfurt/Main, Germany. Data were collected from the first quarter of 2017 until the fourth quarter of 2017. The ethics commission of Goethe University Frankfurt gave its approval to this study.

Inclusion criteria were unilateral CI treatment at least 1 year and a maximum of 10 years ago, more than 65 years of age (at the time of the survey), German language skills at native speaker level. Exclusion criteria were known dementia or other mental illness (depression, psychosis).

Initially, all patients treated at the Department of Oto-Rhino-Laryngology,  University Hospital Frankfurt, who fit the inclusion criteria were informed about the study by telephone. Among these patients, seven could not be contacted and one patient declined to participate in the study. The questionnaire was sent to all remaining patients by mail. The patients completed a questionnaire on quality of life (WHOQL-OLD) in addition to demographic data.

To better assess the development of the collected parameters over time, the study cohort was divided into three groups:

- Group I: patients who were treated with a CI between one and 3 years ago
- Group II: patients who were treated with a CI four to 6 years ago
- Group III: patients who were treated with a CI seven to 10 years ago

In addition, the data from this study were compared with the results of our previous study (Issing et al. [22]) and the normative baseline scores of an average age population according to Conrad et al. [24].

### Patients

In total, questionnaires were sent to 93 patients. Of these, however, nine patients had to be excluded because either the questionnaire was not returned (*n* = 6) or the questionnaire was answered incompletely (*n* = 3). The study thus included 84 patients (36 men and 48 women) between the ages of 65 and 101 years. The average age at the time of the survey was 75.3 ± 7.3 years. At the time of implantation, the average age was 70.4 ± 7.3 years. The total cohort was divided into three groups as described above. All candidates had profound unilateral or bilateral hearing loss and had been treated with a CI unilaterally for at least 1 year and a maximum of 10 years at the time of the survey.

### Freiburg monosyllabic speech test

In addition, audiological data collected during clinical routine preoperatively and 1 year postoperatively were analyzed. The Freiburg monosyllabic speech test (FMS) was used for all patients preoperatively and 1 year after implantation to determine the monosyllable recognition in free field. The non-CI ear was masked by broadband noise or mechanical blocking. The measurement was conducted in best-aided condition preoperatively and postoperatively with a CI at 65 dB SPL.

### Quality of life assessment

The aim of the study was to assess quality of life in older patientes undergoing hearing rehabilitation with respect to the time intervall since CI-fitting was initiated. For the standardized assessment of quality of life, the German version of the World Health Organization Quality-of-Life Scale–old (WHOQL-OLD) was used according to Conrad et al. [24].

This questionnaire, specially developed for patients over the age of 60, takes particular account of the multidimensionality of the quality of life. Six dimensions of the quality of life, so-called facets, are covered:

#### "Sensory abilities"

This facet generally represents the sensory functions (such as hearing, seeing, or tasting) [24].

#### "Autonomy"

"Autonomy" captures the ability to live a self-determined, independent life [24].

#### "Past, present and future activities"

This facet represents achievements already accomplished in life, ongoing activities, and those planned for the future [24].

#### "Social participation"

Participation in social life and social interactions are queried here [24].

#### "Death and Dying"

In addition to concerns about one's own death, this facet also considered the loss of nearby relatives [24].

#### "Intimacy"

The facet "intimacy" describes the importance of human relationships [24].

### Data analysis and statistical evaluation

Data extraction and transfer of the paper-based questionnaires were performed using Microsoft Excel 2016 (Microsoft Corporation, Redmond, Washington). The statistic programs BiAS 11.06 (epsilon-Verlag Hochheim Darmstadt) and GraphPad Prism Version 9 (GraphPad Software, Inc. San Diego) were used for statistical evaluation of the data and application of the statistical test procedures.

First, the Shapiro–Wilk test was used to test for normal distribution. In the absence of a normal distribution, non-parametric tests were used. The Kruskal–Wallis test was used for group comparisons. For the comparisons of our study cohort with the data of the average elderly population, according to Conrad et al. [24], the Wilcoxon-matched pairs test was used. The significance level was set at *p* ≤ 0.05.

## Results

This prospective cross-sectional study included 84 patients aged 65 years and older treated unilaterally with a CI between 1 and 10 years ago. Implants from manufacturers Advanced-Bionics (Advanced-Bionics: Sonova Holding AG, Stäfa, Switzerland) (2.4%; *n* = 2), Cochlear (Cochlear: Cochlear Ltd., Macquarie, Australia) (47.6%; *n* = 40) and Med-EL (MED-EL Elektromedizinische Geräte, Gesellschaft m.b.H., Innsbruck, Austria) (*n* = 50.0%; *n* = 42) were used.

To enable a statement on the development of the different parameters after several years post CI treatment, the patients were divided into three groups:

Group I (1–3 years after CI treatment)

We included 31 patients (15 men and 16 women) with a mean age of 75.0 ± 8.3 years at the time of the survey. The average time the speech processor was worn was reported by 83.9% as more than 12 h per day, and 6.5% in group I wore the speech processor between 6 and 12 h.

Group II (4–6 years after CI treatment)

Group II included 40 patients (19 men and 21 women) with a mean age of 75.2 ± 7.2 years. The wearing time of the speech processor was 70% over 12 h and 20% 6—12 h.

Group III (7–10 years after CI treatment)

In this group, there were 13 patients (2 men and 11 women) with a mean age of 76.7 ± 4.4 years. Regarding this group, 66.7% of patients wore the speech processor for more than 12 h, and 33.3% wore it for 6—12 h.

### Freiburg monosyllabic speech test (FMS)

In best-aided condition, preoperative monosyllabic discrimination in the ear to be treated with a CI was on average at 65 dB SPL in group I 15.3 ± 19.3%, in group II 16.9 ± 24.7% and 9.6 ± 12.3% in group III. The monosyllabic discrimination increased 1 year after implantation at 65 dB SPL to 68.0 ± 19.7% in group I, to 68.0 ± 28.2% in group II, and to 55.4 ± 21.9% in group III. In all three groups, the increase in monosyllabic discrimination from preoperative measurement to follow-up at 1 year was significant (*p* < 0.001). There was no significant difference between the three groups either preoperatively (*p* = 0.956) or 1 year postoperatively (*p* = 0.112).

### World Health Organization Quality-of-Life Scale-Old (WHOQL-OLD)

The WHOQOL-OLD questionnaire measures a total score and six facets of quality of life. In Table 1, in addition to the data of this study, the data of our previous study (preoperative and 6 months postoperative) [22] as well as data of an age-matched average population from Conrad et al. [24] are shown.

**Table 1:** Overview of total WHOQOL-OLD score and the individual facets

	Preoperative (Issing et al. [22])	6 months postoperative (Issing et al. [22])	Group I (1–3 years postoperative)	Group II (4–6 years postoperative)	Group III (7–10 years postoperative)	Control Group ≥ 60 years (Conrad et al. [24])
Total Score	60.0 ± 15.7	66.8 ± 12.2	67.9 ± 11.1	69.4 ± 10.5	65.7 ± 11.4	68.0 ± 14.7
Sensory Abilities	38.1 ± 22.6	57.9 ± 12.6	54.8 ± 18.5	53.5 ± 15.6	54.5 ± 20.8	75.85 ± 21.1
Autonomy	63.2 ± 17.6	65.3 ± 15.3	74.1 ± 15.8	71.7 ± 16.8	68.1 ± 19.1	68.9 ± 19.1
Past, Present and Future Activities	66.2 ± 18.0	68.4 ± 13.8	69.2 ± 15.8	73.1 ± 15.5	75.8 ± 14.5	65.34 ± 16.7
Social Participation	61.04 ± 21.0	70.6 ± 13.6	67.1 ± 17.5	72.0 ± 11.3	65.9 ± 17.6	69.0 ± 20.0
Death and Dying	61.9 ± 30.0	65.6 ± 25.1	69.0 ± 24.1	71.2 ± 19.4	56.3 ± 21.7	62.91 ± 24.3
Intimacy	69.3 ± 20.2	73.0 ± 16.3	72.3 ± 17.0	73.6 ± 16.4	76.0 ± 17.5	65.81 ± 20.9

Complementary are the data preoperatively and 6 months postoperatively of our previous study (Issing et al. [22]). In addition, the normal values of an age-adjusted control group according to Conrad et al. [24] are shown

For the WHOQOL-OLD total score, the average of group I was 67.9 ± 11.1, group II 69.4 ± 10.5, and group III 65.7 ± 11.4 points. There was no significant difference between the three groups (*p* = 0.487).

#### WHOQL-OLD "Sensory abilities"

On this facet, in average group I scored 54.8 ± 18.5, group II 53.5 ± 15.6, and group III 54.5 ± 20.8 points. No significant difference could be found between the three groups (*p* = 0.942).

#### WHOQL-OLD "Autonomy"

For autonomy, group I scored 74.1 ± 15.8, group II 71.7 ± 16.8, and group III 68.1 ± 19.1 points. There was no significant difference between the three groups (*p* = 0.522).

#### WHOQL-OLD "Past, present and future activities"

Regarding this facet, group I scored 69.2 ± 15.8, group II scored 73.1 ± 15.5, and group III scored 75.8 ± 14.5 points. Between the three individual groups, there was no significant difference (*p* = 0.384).

#### WHOQL-OLD "Social participation"

Group I could measure 67.1 ± 17.5, group II 72.0 ± 11.3, and group III 65.9 ± 17.6 points. There was no significant difference (*p* = 0.645) between the three groups.

#### WHOQL-OLD "Death and dying"

In "Death and Dying," group I had 69.0 ± 24.1 points, group II had 71.2 ± 19.4 points and group III had 56.3 ± 21.7 points. There was no significant difference between the three groups (*p* = 0.127).

#### WHOQL-OLD "Intimacy"

On this facet, group I achieved a score of 72.3 ± 17.0, group II of 73.6 ± 16.4, and group III of 76.0 ± 17.5. Again, there was no significant difference between the three groups (*p* = 0.646).

## Discussion

In recent years, the percentage of CI candidates over 65 years of age has steadily risen in many CI centers. Because the expectations for speech understanding from CI treatment are similar to those for younger patients, CI treatment is provided in many countries with no age limit for suitable patients [5, 8, 9, 11]. In addition to a pure audiological assessment, further aspects of hearing rehabilitation, such as the quality of life, are gaining importance as a measure of treatment success. There is strong evidence in the literature for a rapid and significant improvement in quality of life with hearing rehabilitation. However, it is largely unknown whether this is a consistent long-term improvement. This study is a follow-up to our previously published study (Issing et al. [22]) in which we focused on the first 6 months after CI treatment.

To assess the quality of life, we chose the WHOQOL-OLD (World Health Organization Quality of Life-OLD) questionnaire [24]. This questionnaire, which was developed specifically for patients aged 60 and older, is characterized by its multidimensional approach to represent the quality of life accurately. Therefore, in addition to a total score, six different so-called facets of quality of life ("Sensory Abilities," "Autonomy," "Past, Present and Future Activities," "Social Participation," "Death and Dying" and "Intimacy") were used to best represent the different aspects of quality of life. Other commonly used questionnaires are generally not validated for this older age group. On the other hand, this questionnaire measures the quality of life multidimensionally but not disease-specifically like other often used questionnaires as the Nijmegen cochlear implant questionnaire, which can be used meaningfully only in patients with hearing impairment [25]. As a result, data from these disease-specific questionnaires cannot be compared with data from the general population. However, the goal of rehabilitation, and thus also of hearing rehabilitation, should aim at the average population of the same age as good as possible. Consequently, comparisons with an average population appear essential.

**Fig. 1:**
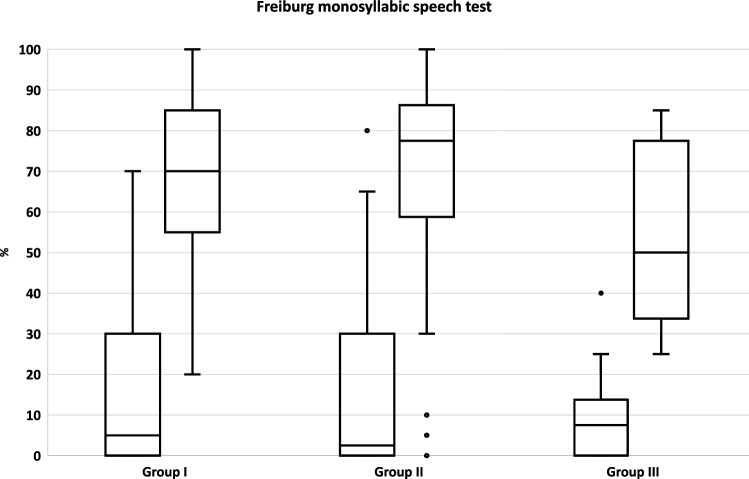
Freiburg monosyllabic speech test (FMS). Results of FMS of the three groups (Group I 1–3 years after CI treatment, Group II 4–6 years after CI treatment, and Group III 7–10 years after CI treatment) preoperatively and one year postoperatively. Preoperative FMS was measured in the ear to be treated with a CI in best-aided condition (contralateral ear blocked or masked). The treated ear was assessed in CI-only condition after 12 months. In all three groups, the increase from preoperative measurement to follow-up at 1 year was significant (*p* < 0.001). At both time points, there was no significant difference between the three groups (preoperatively *p* = 0.956; postoperatively *p* = 0.112).

To reliably measure the potential change of the quality of life over time, the study cohort was divided into three comparable groups (group I 1–3 years after CI treatment, group II 4–6 years after CI treatment, and group III 7–10 years after CI treatment). In addition to the mean age at implantation (group I 72.2 ± 8.3, group II 69.9 ± 7.1 and group III 67.7 ± 4.0 years), the audiological findings preoperatively and 1 year postoperatively were comparable (see Fig. 1). As result, there were no significant differences between the three groups in the Freiburg monosyllabic speech test (FMS) (preoperatively *p* = 0.956; 1 year postoperatively *p* = 0.112). Thus, all three groups showed similar audiological benefits from hearing rehabilitation with CI. This allows standardized comparisons of quality of life between the three groups, excluding possible confounding variables, such as different audiological outcomes or different ages.

**Fig. 2:**
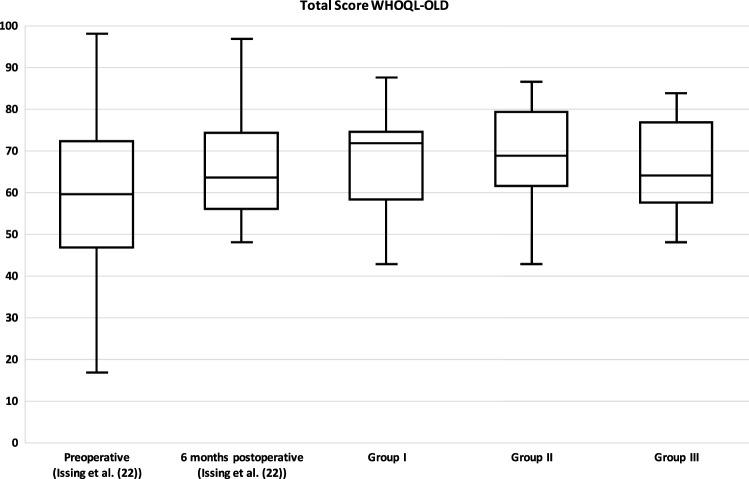
Total WHOQOL-OLD score. The total score is formed from the six individual facets shown in Fig. 3. There were no significant differences between the three groups in the WHOQOL-OLD total score (*p* = 0.487). Complementary results preoperatively and 6 months postoperatively from our previous study (Issing et al. [22]) are presented

**Fig. 3:**
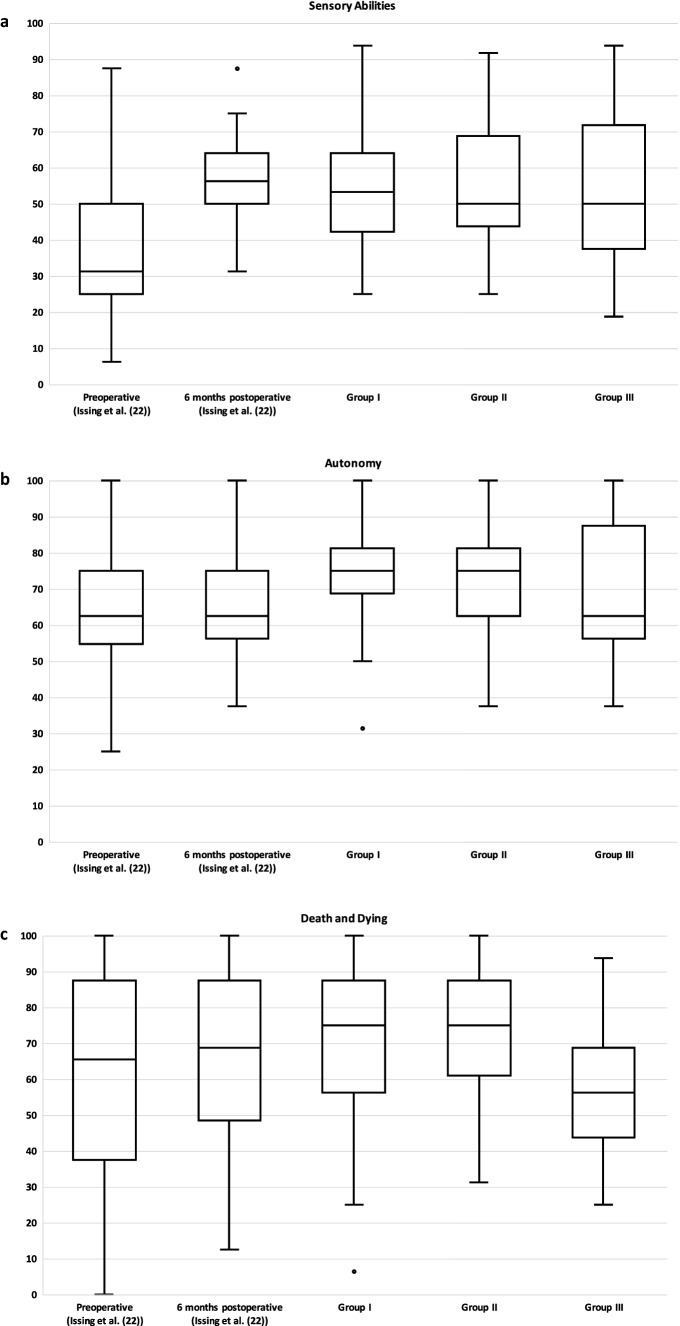

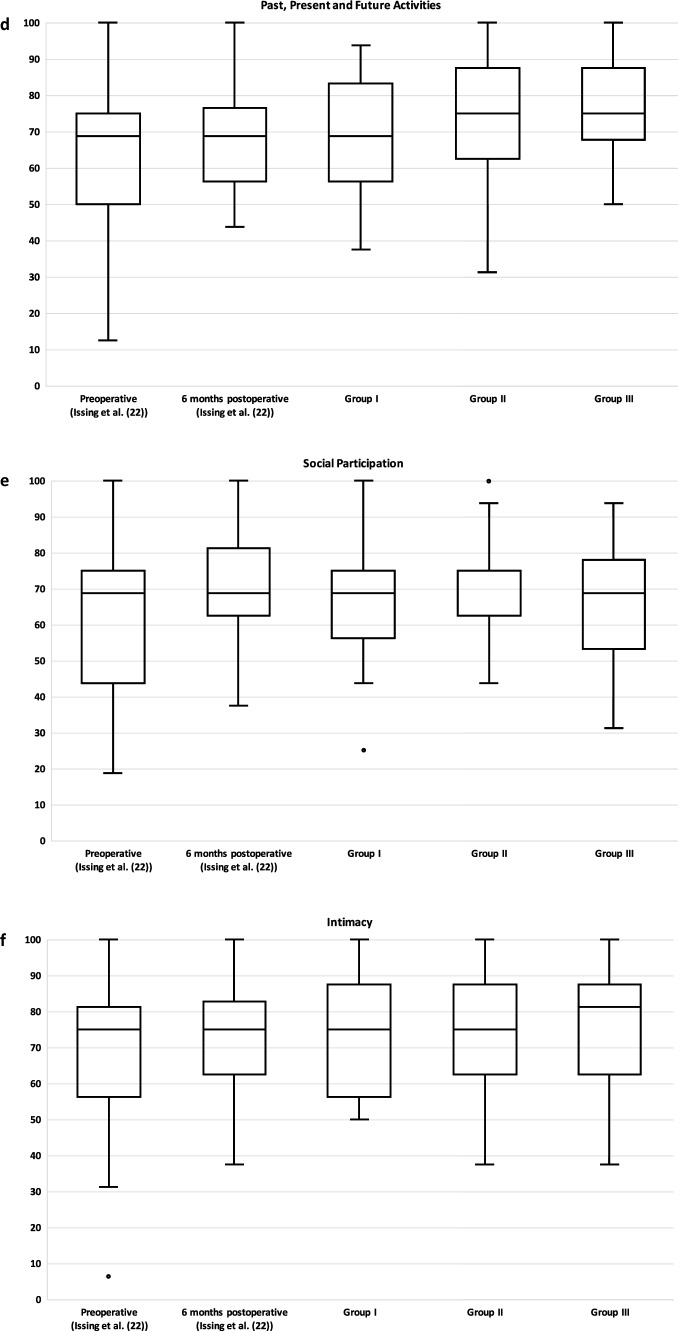
Facets of WHOQOL-OLD. In (**a**–**f**), the individual facets of WHOQOL-OLD and their scores in points (0–100) of the three groups are shown. **a** “Sensory Abilities”; **b** "Autonomy"; **c** "Past, Present and Future Activities"; **d** "Social Participation"; **e** "Death and Dying"; **f** "Intimacy." There was no significant difference between the three groups in any facet (*p* > 0.05). Complementary results preoperatively and 6 months postoperatively from our previous study (Issing et al. [22]) are presented

Our primary goal for this study was to evaluate the long-term effects of hearing rehabilitation in elderly patients aged 65 and older. Considering the WHOQL-OLD total score, our results showed no significant difference between the three groups. Also, when looking at the individual facets there were no significant differences. Comparing the results of this study with the data 6 months postoperatively from our previous study [22], in which we examined only the first 6 months after CI treatment, there were no significant differences neither in the total score (*p* = 0.529) nor in the individual facets (“Sensory abilities” *p* = 0.556; “Autonomy” *p* = 0.078; “Past, Present and Future Activities” *p* = 0.21; “Social Participation” *p* = 0.812; “Death and Dying” *p* = 0.256; “Intimacy *p* = 0.802) for any of the three groups (see Figs. 2 and 3A–F). Consequently, our data of this study compared with the data of our previous study [22] indicate a long-term stable improvement in quality of life over the years not only in the total score but also when looking at all individual facets. Elderly patients thus seem to show a significant improvement in quality of life already about 6 months after hearing rehabilitation by CI and then keep this level stable for years. In the literature, mainly only the short- and medium-term positive effects of CI treatment on quality of life have been described so far [19–23, 26–29].

These undoubtedly positive effects of CI treatment should also be considered in the context of an age-adjusted average population. Comparing the "sensory abilities" of our three groups (Group I 54.8 ± 18.5; Group II 53.5 1 ± 5.6 and Group III 54.5 ± 20.8 points) with the score of the age-adjusted average population of 75.85 ± 21.1 (24), all three groups showed a significant difference (Group I *p* = 0.001; Group II *p* = 0.001; Group III *p* = 0.005). So even after years, the patients treated with a CI do not reach the level of the average population in terms of "sensory abilities". When comparing the total score of the three groups with the score of the average population [24], there is no significant difference (Group I *p* = 0.97; Group II *p* = 0.336; Group III *p* = 0.47).

A critical review of the study reveals potential limitations: The study design did not have a control group. Instead, normative values of an age-adjusted average population from the literature had to be used [24]. Second, this was no proper longitudinal study design, in which the same patient is followed multiple times over the period of treatment. Instead, we interviewed patients who had been implanted for different lengths of time at one timepoint. This was partly because many older patients, in particular, do not usually attend the annual CI check-up appointments, e.g., due to other health problems, and therefore often present themselves irregular to CI consultation. Although it is unlikely to influence the results, it should be noted that in group III the gender distribution (2 men and 11 women) is not balanced.

Further randomized long-term studies are therefore needed to evaluate the long-term effects more extensively.

In summary, our data of this study compared with the results of our previous study [22] demonstrate a stable improvement in quality of life over many years after CI treatment and therefore emphasize the benefit of CI treatment in the elderly population. This is a valuable result to be presented to patients before CI surgery to help the decision-making process.

## Conclusion

The results our study demonstrate the positive long-term effect on the improvement in quality of life resulting from hearing rehabilitation using CI in patients aged 65 years and older. There was no significant deterioration in the WHOQOL-OLD total score or in any of the six facets. Nevertheless, despite CI treatment, the patients did not reach the level of the average population in "sensory abilities" even after years.

## References

[1] Lin FR, Niparko JK, Ferrucci L (2011). Hearing loss prevalence in the United States. Arch Intern Med.

[2] Lin FR, Thorpe R, Gordon-Salant S, Ferrucci L (2011). Hearing loss prevalence and risk factors among older adults in the United States. J Gerontol A Biol Sci Med Sci.

[3] Rechel B, Grundy E, Robine JM, Cylus J, Mackenbach JP, Knai C, McKee M (2013). Ageing in the European Union. Lancet.

[4] Manrique-Huarte R, Calavia D, Huarte Irujo A, Giron L, Manrique-Rodriguez M (2016). Treatment for hearing loss among the elderly: auditory outcomes and impact on quality of life. Audiol Neurootol.

[5] Chatelin V, Kim EJ, Driscoll C, Larky J, Polite C, Price L, Lalwani AK (2004). Cochlear implant outcomes in the elderly. Otol Neurotol.

[6] Kempf HG, Büchner A, Stöver T (2003). Cochlea implants in adults: indications and realization. Special cases and technical parameters of the implantation systems [in German]. HNO.

[7] Wong DJ, Moran M, O'Leary SJ (2016). Outcomes After Cochlear Implantation in the Very Elderly. Otol Neurotol.

[8] Dillon MT, Buss E, Adunka MC, King ER, Pillsbury HC, Adunka OF, Buchman CA (2013). Long-term speech perception in elderly cochlear implant users. JAMA Otolaryngol Head Neck Surg.

[9] Buchman CA, Fucci MJ, Luxford WM (1999). Cochlear implants in the geriatric population: benefits outweigh risks. Ear Nose Throat J.

[10] Ghiselli S, Nedic S, Montino S, Astolfi L, Bovo R (2016). Cochlear implantation in post-lingually deafened adults and elderly patients: analysis of audiometric and speech perception outcomes during the first year of use. Acta Otorhinolaryngol Ital.

[11] Hilly O, Hwang E, Smith L, Shipp D, Nedzelski JM, Chen JM, Lin VW (2016). Cochlear implantation in elderly patients: stability of outcome over time. J Laryngol Otol.

[12] Lin FR, Yaffe K, Xia J, Xue QL, Harris TB, Purchase-Helzner E, Satterfield S, Ayonayon HN, Ferrucci L, Simonsick EM (2013). Hearing loss and cognitive decline in older adults. JAMA Intern Med.

[13] Issing C, Baumann U, Pantel J, Stöver T (2021). Impact of hearing rehabilitation using cochlear implants on cognitive function in older patients. Otol Neurotol.

[14] Jayakody DMP, Friedland PL, Nel E, Martins RN, Atlas MD, Sohrabi HR (2017). Impact of cochlear implantation on cognitive functions of older adults: pilot test results. Otol Neurotol.

[15] Mosnier I, Bebear J-P, Marx M, Fraysse B, Truy E, Lina-Granade G, Mondain M, Sterkers-Artières F, Bordure P, Robier A, Godey B, Meyer B, Frachet B, Poncet-Wallet C, Bouccara D, Sterkers O (2015). Improvement of cognitive function after cochlear implantation in elderly patients. JAMA Otolaryngol.

[16] Sarant J, Harris D, Busby P, Maruff P, Schembri A, Dowell R, Briggs R (2019). The effect of cochlear implants on cognitive function in older adults: initial baseline and 18-month follow up results for a prospective international longitudinal study. Front Neurosci.

[17] Mick P, Kawachi I, Lin FR (2014). The association between hearing loss and social isolation in older adults. Otolaryngol Head Neck Surg.

[18] Arlinger S (2003). Negative consequences of uncorrected hearing loss-a review. Int J Audiol.

[19] Dalton DS, Cruickshanks KJ, Klein BE, Klein R, Wiley TL, Nondahl DM (2003). The impact of hearing loss on quality of life in older adults. Gerontologist.

[20] Olze H, Szczepek AJ, Haupt H, Zirke N, Graebel S, Mazurek B (2012). The impact of cochlear implantation on tinnitus, stress and quality of life in postlingually deafened patients. Audiol Neurootol.

[21] Vermeire K, Brokx JP, Wuyts FL, Cochet E, Hofkens A, Van de Heyning PH (2005). Quality-of-life benefit from cochlear implantation in the elderly. Otol Neurotol.

[22] Issing C, Baumann U, Pantel J, Stöver T (2020). Cochlear implant therapy improves the quality of life in older patients—a prospective evaluation study. Otol Neurotol.

[23] Aimoni C, Ciorba A, Hatzopoulos S, Ramacciotti G, Mazzoli M, Bianchini C, Rosignoli M, Skarżyński H, Skarżyński PH (2016). Cochlear implants in subjects over age 65: quality of life and audiological outcomes. Med Sci Monit.

[24] Conrad I, Matschinger H, Kilian R, Riedel-Heller S (2000). WHOQOL-OLD und WHOQOL-BREF. Handbuch für die deutschsprachigen Versionen der WHO-Instrumente zur Erfassung der Lebensqualität im Alter [in German].

[25] Hinderink JB, Krabbe PF, Van Den Broek P (2000). Development and application of a health-related quality-of-life instrument for adults with cochlear implants: the Nijmegen cochlear implant questionnaire. Otolaryngol Head Neck Surg.

[26] Knopke S, Grabel S, Forster-Ruhrmann U, Mazurek B, Szczepek AJ, Olze H (2016). Impact of cochlear implantation on quality of life and mental comorbidity in patients aged 80 years. Laryngoscope.

[27] Yang Z, Cosetti M (2016). Safety and outcomes of cochlear implantation in the elderly: a review of recent literature. J Otol.

[28] Sonnet MH, Montaut-Verient B, Niemier JY, Hoen M, Ribeyre L, Parietti-Winkler C (2017). Cognitive abilities and quality of life after cochlear implantation in the elderly. Otolo Neurotol.

[29] Wick CC, Kallogjeri D, McJunkin JL, Durakovic N, Holden LK, Herzog JA, Firszt JB, Buchman CA, Group CS (2020). Hearing and quality-of-life outcomes after cochlear implantation in adult hearing aid users 65 years or older: a secondary analysis of a nonrandomized clinical trial. JAMA Otolaryngol Head Neck Surg.

